# Comparison of identification of sentinel lymph nodes between ICG vs methylene blue in v notes staging surgery for endometrial cancer

**DOI:** 10.1186/s12893-025-03232-w

**Published:** 2025-10-21

**Authors:** Kevser Arkan, Ali Deniz Erkmen, Mesut Ali Haliscelik, Seyhmus Tunc, Gul Cavusoglu Colak, Sedat Akgol, Behzat Can

**Affiliations:** 1 Department Obstetrics and Gynecology, Division of Gynecologic Oncology, Diyarbakir Gazi Yasargil Research and Training Hospital, Elazig Road 10.Km Uckuyular, Diyarbakir, Kayapinar 21070 Turkey; 2 Department Obstetrics and Gynecology, Division of Gynecologic Oncology, Bower Hospital, Diyarbakir, Turkey

**Keywords:** Endometrial cancer, Sentinel lymph node biopsy, Indocyanine green, Methylene blue, V-NOTES, Minimally invasive surgery

## Abstract

**Objective:**

This study aimed to assess the comparative effectiveness of indocyanine green and methylene blue in the marking and identification of sentinel lymph nodes during sentinel lymph node biopsy procedures in patients diagnosed with endometrial cancer undergoing staging surgery through vaginal natural-orifice transluminal endoscopic surgery.

**Methods:**

In this retrospective cohort study conducted at a tertiary center, we analyzed 80 patients with endometrial cancer who underwent vaginal natural orifice transluminal endoscopic staging surgery. Patients were classified into two cohorts based on the tracer used for sentinel lymph node sentinel lymph node mapping: indocyanine green (*n* = 40) or methylene blue (*n* = 40). The primary endpoints, including sentinel lymph node detection rates (overall and bilateral) and number of nodes retrieved, along with surgical outcomes, were compared between the groups.

**Findings:**

: Eighty patients (indocyanine green group, *n* = 40; methylene blue group, *n* = 40) were included in the study. The vaginal natural orifice transluminal endoscopic surgery identification rate was significantly higher in the indocyanine green group (95%) than in the methylene blue group (82.5%) (*p* = 0.045). The mean number of sentinel lymph nodes identified in the indocyanine green group (3.2 ± 1.1) was significantly higher than that in the methylene blue group (2.5 ± 0.9) (*p* = 0.021). The rate of bilateral vaginal natural orifice transluminal endoscopic surgery identification was higher in the indocyanine green group (80%) than in the methylene blue group (65%); however, the difference was not statistically significant (*p* = 0.112). Surgical time, blood loss, and complication rates were similar between the two groups. Histopathological examination revealed a similar number of positive sentinel lymph nodes in both the groups.

**Conclusion:**

In the context of sentinel lymph node biopsy for staging surgery and natural orifice transluminal endoscopic surgery for endometrial cancer, indocyanine green has demonstrated a superior sentinel lymph node identification rate and a higher yield of sentinel lymph nodes compared to methylene blue. Given the advantage of real-time imaging, indocyanine green has emerged as a promising agent for sentinel lymph node biopsy in minimally invasive approaches, such as vaginal natural orifice transluminal endoscopic surgery.

## KEY MESSAGE

**What is already known?:**

Minimally invasive surgery and SLNB are feasible and safe for the treatment of endometrial cancer. ICG and methylene blue are SLN tracers.

**What this study adds?:**

ICG yielded significantly higher SLN identification and more SLNs than methylene blue in V-NOTES staging for endometrial cancer.

**How might this affect research, practice or policy?:**

ICG shows promise as a superior SLN tracer in V-NOTES for endometrial cancer staging, warranting larger trials to optimize protocols.

## Introduction

 Endometrial cancer (EC) is the most common gynecological cancer in developed countries, and staging is essential to facilitate treatment recommendations for adjuvant therapy [1]. The use of sentinel lymph node (SLN) biopsy as part of minimally invasive staging surgery provides reliable lymph node assessment and significantly lower morbidity compared to lymphadenectomy [2, 3]. SLN mapping has less morbidity than traditional extended pelvic and paraaortic lymphadenectomy and has comparable oncological outcomes with significantly lower rates of lymphedema and intraoperative complications [4]. The concept behind the SLN method is based on the premise that tumor cells metastasize in a predictable manner stepwise to the SLN prior to arriving at an upper-level lymph node. This method allows for selective dissection of regional lymph nodes, providing a safer surgical alternative with potentially lower morbidity [5]. Methods for SLN detection usually involve tracers of indocyanine green (ICG) or methylene blue (MB). Indocyanine green (ICG) provides near-infrared fluorescence imaging of lymphatic drainage and higher SLN detection rates in minimally invasive procedures [6, 7]. Methylene blue (MB) is a cheaper dye that is easily accessible with purposeful visual staining of the lymphatics and does not offer a means of real-time imaging [1, 8].

Vaginal natural orifice transluminal endoscopic surgery (V-NOTES) is an innovative, incisionless surgical approach that has been successfully adapted for various gynecological procedures including hysterectomy and staging surgeries for endometrial cancer [9, 10]. Pioneering work by groups such as Lee et al. demonstrated the feasibility of this technique across different uterine sizes and for complex procedures, such as sentinel lymph node dissection [11, 12]. Given that this approach can provide excellent visualization and direct access to retroperitoneal structures, V-NOTES has the potential to improve SLN detection rates while minimizing surgical trauma [13].

However, there is a lack of data on the use of dye agents that sequentially compare SLN mapping effectiveness with SLN mapping, leaving us uncertain as to which agent is best for SLN mapping in the specific context of V-NOTES.

Consequently, we aimed to conduct a prospective study to compare the reliability of ICG and MB for SLN mapping in EC patients undergoing V-NOTES staging surgery with respect to SLN detection rates, accuracy, and clinical outcomes.

## Materials and methods

### Study approval and design

This retrospective study was conducted in accordance with the Declaration of Helsinki. The study protocol was approved by the Ethics Committee of Gazi Yasargil Training and Research Hospital, Diyarbakır, Turkey (Approval No. 169, Date: 13/09/2024). The requirement for written informed consent was waived by the ethics committee, in accordance with national regulations, due to the retrospective design and the use of fully anonymized patient data.The study adhered to the principles outlined in the Helsinki Declaration and the STROBE reporting guidelines.

### Study design and patient selection

This retrospective cohort controlled trial was conducted in accordance with the Declaration of Helsinki. The study protocol was approved by the Gazi Yasargil Training and Research Hospital Clinical Research Ethics Committee (Decision No: 169, Date: 13.09.2024).

Due to the retrospective nature of the study, which involved the analysis of pre-existing and anonymized patient data, the requirement for obtaining individual informed consent was formally waived by the ethics committee. Patient data confidentiality was maintained throughout the study.

The study included consecutive patients with newly diagnosed endometrial cancer. Patients were classified as FIGO stage I-II and were scheduled to undergo total hysterectomy, bilateral salpingo-oophorectomy (BSO), and SLN biopsy using the V-NOTES approach.

Patients with suspected metastatic disease, prior pelvic or para-aortic lymphadenectomy, known allergies, or hypersensitivity to the marking agents indocyanine ICG or MB were excluded from the study.

The final analysis included 80 patients who successfully underwent complete V-NOTES staging. Cases that required conversion to standard laparoscopy (*n* = 12), due to reasons such as the need for para-aortic lymphadenectomy or significant intraoperative complications, were not included in this comparative analysis, as the primary objective was to evaluate the performance of the dyes within the specific context of the V-NOTES procedure.

### Application of marking agents

#### ICG group

After induction of anesthesia, a 1.25 mg/mL solution of ICG was prepared by diluting a vial of ICG (VerDye^®^, Akorn Pharmaceuticals) containing 25 MG in 20 mL of sterile water. A total of 4–5 mL of this solution was injected into the cervix at the three and nine o’clock positions. The injection protocol was as follows:

##### Stromal injection

1 mL depth of 1 cm on each side (right and left).

##### Submucosal injection

**Fig. 1:**
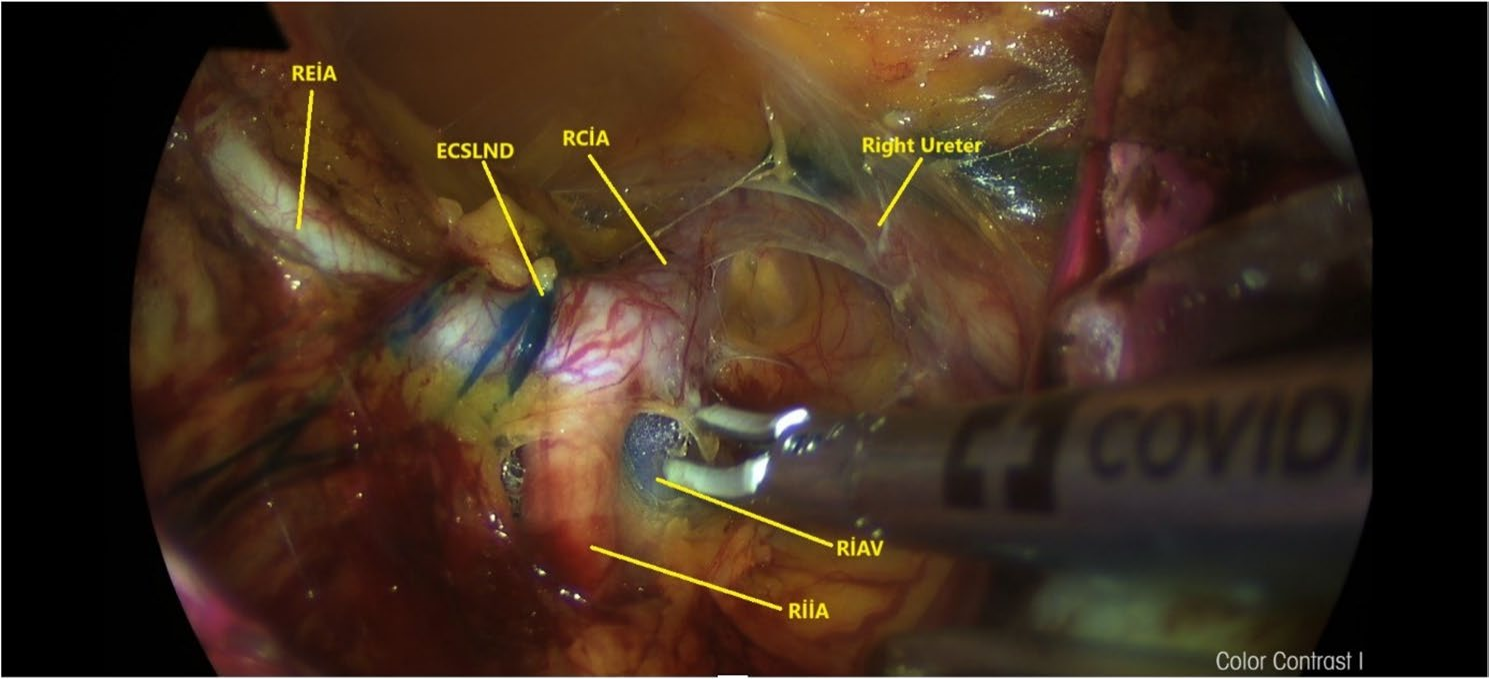
Anatomical view of the right pelvic paravesical area stained with methylene blue following lymph node dissection via retroperitoneal transvaginal approach with vNOTES. (ECSLN: Efferent channels of the sentinel lymph node, RCIA: Right common iliac artery, REIA: Right external iliac artery, REIV: Right external iliac vein,RIIA: Right internal iliac artery, RIIV: Right internal iliac vein, RON: Right obturator nerve, RPM: Right psoas muscle)

1 mL, in the same position and targeting the submucosal area. This injection is generally performed after the initial laparoscopic exploration to demonstrate the timeliness of the surgical process. At this point, SLNs were visualized using a near-infrared fluorescence imaging system (Karl Storz IMAGE1 S™ Rubina® ) in real time to ascertain the proper distribution of the drug (Figure 1).

This injection is generally performed after the initial laparoscopic exploration to demonstrate the timeliness of the surgical process. At this point, SLNs were visualized using a near-infrared fluorescence imaging system (Karl Storz IMAGE1 S™ Rubina^®^) in real time to ascertain the proper distribution of the drug (Fig. 1).

#### MB group

**Fig. 2:**
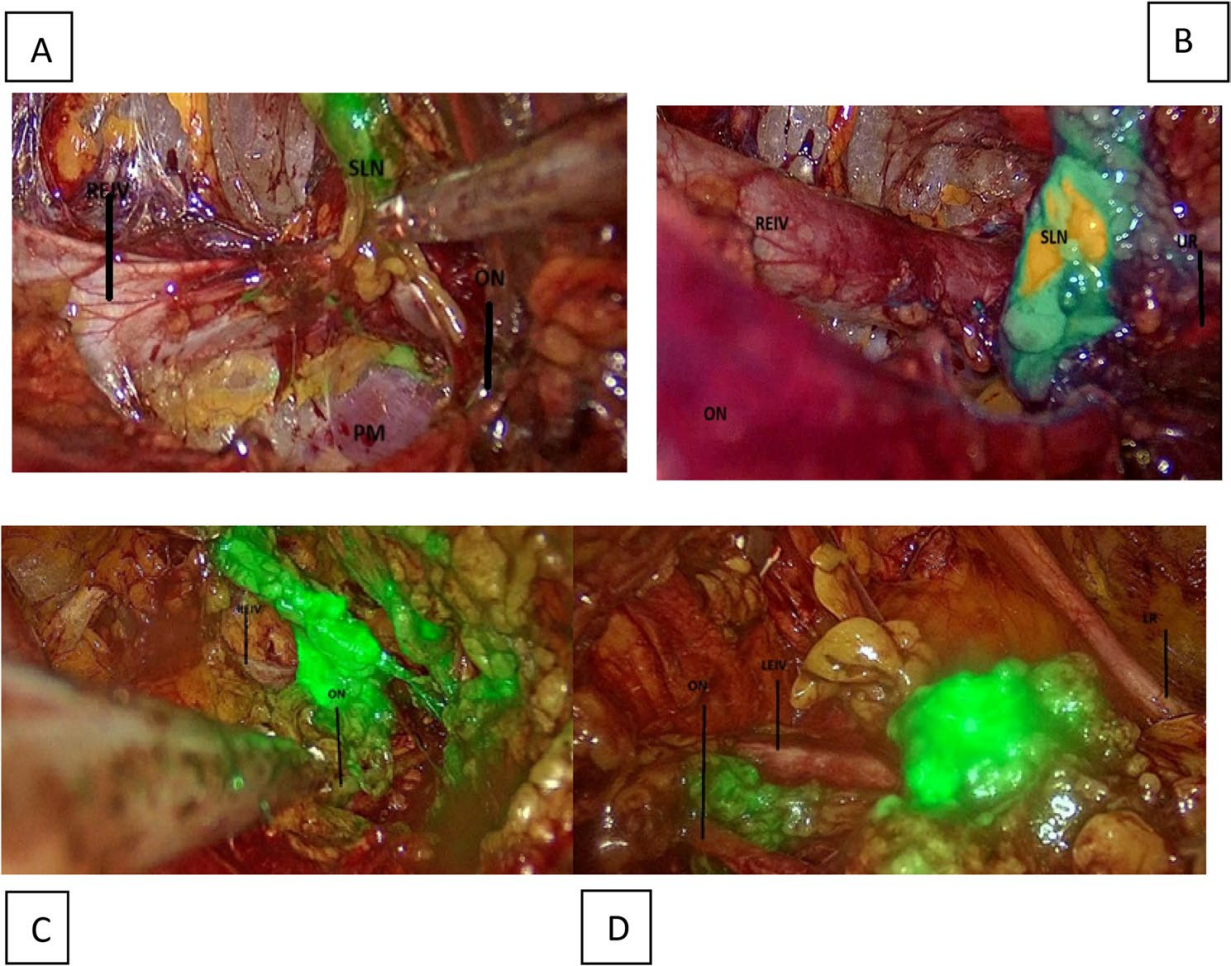
**a-)** Anatomical view of the right pelvic and **b-)** left pelvic paravesical area stained ICG following lymph node dissection via retroperitoneal transvaginal approach with vNOTES. **A**,**B**,**C**: Right pelvic sentinel lymphadenectomy. **D**: Left pelvic lymphadenectomy (extra dissection). REIV, right external iliac vein; ON, obtrurator nerve; LEIV, left externai iliac vein, LR, ligamentum rotundum, UR, ureter

For patients in the MB group, following anesthesia induction, 4 mL of methylene blue dye was administered, 2 mL at the three and nine o’clock positions on the cervix. Injection at the 12 o’clock position was avoided to eliminate the potential for dye migration into the vesicocervicovaginal space and the added danger of complicated dissection of the bladder. To allow for ideal transport of the dye, the surgical process commenced approximately 15 min after cervical injection (Fig. 2).

### Surgery

All the patients included in the study underwent a comprehensive preoperative evaluation. As a standard protocol, whole-body PET-CT was performed for every patient to assess the extent of the disease and exclude distant metastasis. Patients with radiological evidence of metastatic disease (M1) on PET/CT were excluded from the study.

All surgical procedures were conducted according to the Memorial Sloan Kettering Cancer Center standardized procedures [14]. After identifying the SLNs, they were excised, individually tagged, and sent to the pathology laboratory for histopathological evaluation.

If no SLNs were identified or there were suspicious or enlarged lymph nodes, then a side-specific or complete pelvic and/or para-aortic lymphadenectomy was performed at the operating surgeon’s discretion based on intraoperative findings and clinical judgment.

### SLN mapping

The patients were placed in the lithotomy position under general anesthesia. The cervix was held and gently drawn to optimize exposure to the injection sites. In both ICG and MB groups, submucosal injection was conducted at the 3 and 9 o’clock positions of the cervix with the addition of 1 mL of either ICG or MB, followed by injection of 1 mL of either at 10 mm depths on either side to facilitate the staining of both deep and superficial lymphatics.

In the MB group, a delay of 15 min was observed following the injection of the dye to allow further uptake and staining of the SLN. For the ICG group, there was no wait for the dye to rapidly travel through the lymph vessels in the body and, consequently, may stain non-targeted tissues if it was not injected quickly because the surgery was delayed.

During the injection phase, the spread of the dye was observed in fine branches in the submucosal vessels, indicating uptake by lymphatics, marking of nodes by dye, and identification of lymphatic drainage pathways, which were assessed and documented intraoperatively. SLNs were found in recognized anatomical locations in the pelvic region, mainly in the obturator fossa and in the region between the internal and external iliac veins. The left and right SLNs, which appeared to be poorly draining and symmetrically stained, were safely excised.

If obvious unilateral or bilateral nodes were not identified, a side-specific or full pelvic lymphadenectomy procedure was performed. Careful excision was performed for any enlarged or suspicious lymph nodes or if additional secondary SLNs demonstrated any type of methylene blue uptake. All surgical specimens were sent for nodal assessment.

### Lymph node dissection

To begin lymph node dissection, the surgical field was stabilized with a single-tooth tenaculum at the entrance of the pelvic retroperitoneal space. To provide sufficient countertraction, the right paracervical vaginal mucosa was grasped approximately 3 cm lateral to the cervix, while an additional instrument provided lateral tension approximately 1 cm from the first grasping point.

We made a 2 cm vertical vaginal mucosal incision at the 9 o’clock position, noting that the dissection plane was kept inferior to minimize the risk of bladder injury. Using blunt dissection with the surgeon’s index finger, we moved through the paracervical connective tissue to gain access to the obturator fossa, maintaining a more medial path relative to the ischial part of the pubic bone to avoid neurovascular injury.

Upon entering the obturator fossa, the vNOTES port was placed, specifically a 7 cm GelPoint V-Path Transvaginal Access Platform (Applied Medical, Rancho Santa Margarita, CA, USA) while providing CO₂ insufflation at 12 mmHg for improved visualization of the retroperitoneal space. A 30-degree endoscope was introduced through a 10-mm trocar and 10 mm trocars were used for bipolar graspers and a bipolar sealing device to avoid excessive energy-based instrument use along the peritoneum, where possible.

We first identified critical anatomical landmarks with the obliterated umbilical artery, external iliac vein, ureter, iliac bifurcation, and internal and external iliac arteries and veins, and found the obturator nerve. We carefully traced the cervical afferent lymphatic ducts to allow dissection of the full extent of the upper paracervical lymphatic drainage region, which included the internal and external iliac and obturator areas. SLNs were excised using advanced bipolar instruments (LigaSure, Voyant) and extracted transvaginally for pathological analysis.

If we did not identify an SLN or had a suspicious and/or enlarged lymph node, we performed a side-specific pelvic lymphadenectomy using the same vNOTES technique and explored the contralateral side. If we identified a bulky lymph node on the right side, we typically began with right-sided SLN dissection and performed left-sided dissection for palliation thereafter.

### Hysterectomy and bilateral salpingo-oophorectomy (BSO)

Following retroperitoneal SLN dissection, laparoscopic survey of the peritoneal cavity was performed through the same V-NOTES port before proceeding with hysterectomy. Because all surgical stages, including nodal dissection, peritoneal survey, and hysterectomy, were completed via a single vaginal entry point, the procedure was classified as V-NOTES.

Upon dissection of the SLN, anterior and posterior colpotomies were completed under the assumption of maintaining vesicouterine and rectouterine clefts to ultimately limit the potential for contamination of the peritoneal cavity. The cervix was closed in a double layer with No. 1 Vicryl sutures to limit the risk of tumor spillage into the peritoneal cavity during vNOTES hysterectomy.

Upon completion of cervical closure the 9 cm GelPoint V-Path Transvaginal Access Platform (Applied Medical, Rancho Santa Margarita, CA, USA) was used to confirm entry into the intraperitoneal cavity. Laparoscopy was conducted to explore the abdominal and pelvic cavities for possible adhesions (with the potential to create a problematic domain for laparoscopic vNOTES hysterectomy), peritoneal implants, or any other potential pathological findings.

After confirming that the intraperitoneal space had no significant adhesions or suspicious lesions, it was time to complete vNOTES-assisted type 1 hysterectomy and BSO using standard surgical procedures, which ensured complete removal of the uterus, ovaries, and fallopian tubes, as previously described [15].

### Statistical analysis

Data for this study were sourced from a prospectively maintained institutional database. Clinical and pathologic information was obtained from this prospectively maintained database for the demographics of each patient, tumor characteristics, and surgical outcomes in a structured format. The primary outcome was the success rate of SLN mapping, which was assessed for overall detection of SLN, detection of bilateral SLN, and specific side.

Categorical variables were analyzed using either the chi-square test or Fisher’s exact test, as appropriate. Continuous variables were measured using Student’s t-test in the event of normally distributed data, and the Mann-Whitney U test in cases of non-normal distribution, determined by the Shapiro-Wilk test for normal distribution. Statistical significance was defined as *P* < 0.05. Statistical analyses were performed using the SPSS software (version 25.0, IBM Corp., Armonk, NY, USA).

## Results

Eighty patients were included in the study, with similar demographic and clinical characteristics between the ICG and methylene blue groups, likely minimizing any confounding variables. The mean age was 62.5 ± 8.1 years (ICG) and 61.8 ± 7.5 years (methylene blue) respectively (*p* = 0.672). Body mass index (BMI) was also similar between groups (ICG: 28.7 ± 4.5 kg/m² vs. methylene blue: 29.1 ± 4.2 kg/m², *p* = 0.719). Parity was not significantly different between groups (ICG: 2.1 ± 1.3 vs. methylene blue:2.0 ± 1.2, *p* = 0.805); as well, the post-menopausal proportions were comparable (ICG: 60.0% vs. methylene blue: 62.5%, *p* = 0.784). The proportions of histological types (*p* = 0.553), tumor grades (*p* = 0.611), and FIGO stages (*p* = 0.829) were similarly distributed (Table 1).

**Table 1:** Patient demographic and clinical characteristics

Feature	ICG Group(*n* = 40)	Metilen blue Group (*n* = 40)	*p* value
Age (years, mean ± SD)	62.5 ± 8.1	61.8 ± 7.5	0.672
BMI (kg/m², mean ± SD)	28.7 ± 4.5	29.1 ± 4.2	0.719
Parity (mean ± SD)	2.1 ± 1.3	2.0 ± 1.2	0.805
Postmenopausal (%)	24 (60.0)	25 (62.5)	0.784
Histological Type(%)			0.553
Endometrioid Adenocarcinoma	32 (80.0)	30 (75.0)	
Serous Adenocarcinoma	5 (12.5)	6 (15.0)	
Other	3 (7.5)	4 (10.0)	
Tumor Grade(%)			0.611
1	18 (45.0)	16 (40.0)	
2	15 (37.5)	17 (42.5)	
3	7 (17.5)	7 (17.5)	
FIGO Stage(%)			0.829
I	37 (92.5)	37 (92.5)	
II	3 (7.5)	3 (7.5)	

While investigating the SLN identification rates, it was found that the ICG group had a higher overall SLN identification rate (95.0%) than the methylene blue group (82.5%) (*p* = 0.045). The ICG group also had a higher rate of bilateral SLN identification (80.0%) than the methylene blue group (65.0%); however, this difference was not statistically significant (*p* = 0.112). The average number of SLNs identified per patient was significantly higher in patients receiving ICG (3.2 ± 1.1) than in those receiving methylene blue (2.5 ± 0.9, *p* = 0.021). There were no statistically significant differences in the elapsed time to the first SLN identification (ICG: 8.5 ± 3.2 min vs. methylene blue: 9.1 ± 3.8 min, *p* = 0.417) or total operating room time (ICG: 145 ± 35 min vs. methylene blue: 152 ± 40 min, *p* = 0.318). Similarly, the estimated intraoperative blood loss (ICG: 85 ± 40 mL vs. methylene blue: 92 ± 45 mL, *p* = 0.489), transfusion requirements (ICG: 5.0% vs. methylene blue: 7.5%, *p* = 0.652), and overall complication rates (ICG: 12.5% vs. methylene blue: 15.0%; *p* = 0.721) were similar (Table 2).

**Table 2:** Surgical outcomes and SLN identification rates

Feature	ICG group(*n* = 40)	Metilen Blue Group (*n* = 40)	*p* value
SLN Identification Rate(%)	38 (95.0)	33 (82.5)	0.045
Bilateral SLN Identification Rate(%)	32 (80.0)	26 (65.0)	0.112
Average Number of SLNs Identified	3.2 ± 1.1	2.5 ± 0.9	0.021
Time to First SLN Identification (min)	8.5 ± 3.2	9.1 ± 3.8	0.417
Total Surgical Time (min)	145 ± 35	152 ± 40	0.318
Estimated Blood Loss(mL)	85 ± 40	92 ± 45	0.489
Transfusion Requirement (%)	2 (5.0)	3 (7.5)	0.652
Complication Rate (%)	5 (12.5)	6 (15.0)	0.721

Histopathologic assessment identified SLN metastasis in eight (21.1%) patients in the ICG group and seven (21.2%) patients in the methylene blue group, with no statistically significant difference (*p* = 0.987). In summary, while ICG offers a higher overall rate of SLN identification and a higher average number of SLNs identified per patient than methylene blue, it does not offer a statistically significant earlier time to first SLN identification or an earlier overall surgical time than methylene blue.

In our study cohort, complete lymphadenectomy was performed in 17 (21.3%) patients. The primary reasons for performing a completion lymphadenectomy were failure to detect an SLN or the presence of intraoperatively suspicious or enlarged lymph nodes. A comparative analysis was conducted between patients who underwent SLN biopsy only and those who required complete lymphadenectomy (Table 3). While the baseline demographic characteristics, including age and BMI, were similar between the two groups, we found that the completion lymphadenectomy group had a significantly higher proportion of high-grade (grade 3) tumors (58.8% vs. 6.3%, *p* < 0.001) and non-endometrioid histology (*p* < 0.001).

**Table 3:** Comparison of clinicopathological characteristics between patients undergoing SLN biopsy only and completion lymphadenectomy

Characteristic	SLN Biopsy Only Group (*n* = 63)	Completion Lymphadenectomy Group (*n* = 17)	*p*-value
Age (years, mean ± SD)	62.3 ± 8.0	63.1 ± 7.9	0.735
BMI (kg/m², mean ± SD)	28.8 ± 4.4	29.5 ± 4.3	0.591
Parity (mean ± SD)	2.1 ± 1.2	1.9 ± 1.4	0.610
Postmenopausal, n (%)	39 (61.9)	10 (58.8)	0.802
Histological Type, n (%)			< 0.001
Endometrioid	60 (95.2)	2 (11.8)	
Serous	1 (1.6)	10 (58.8)	
Other	2 (3.2)	5 (29.4)	
Tumor Grade, n (%)			< 0.001
Grade 1	33 (52.4)	1 (5.9)	
Grade 2	26 (41.3)	6 (35.3)	
Grade 3	4 (6.3)	10 (58.8)	
FIGO Stage, n (%)			0.002
Stage I	62 (98.4)	12 (70.6)	
Stage II	1 (1.6)	5 (29.4)	

## Discussion

Lymph node (LN) involvement remains the primary prognostic factor for women diagnosed with endometrial and cervical cancers. However, due to the low incidence of LN metastasis in early stage patients, there has been a significant paradigm shift away from more invasive lymphatic assessment techniques, such as radical pelvic lymphadenectomy or aortic lymphadenectomy, towards less invasive lymphatic assessment techniques (SLN mapping) over the last couple of decades. The V-NOTES surgical approach has become an alternative to conventional laparoscopic techniques, potentially reducing morbidity associated with vertical infertility and complete lymphadenectomy. Unlike traditional laparoscopic techniques that approach the lymphatic basin from the periphery of the body, V-NOTES can target the primary lymph nodes directly from the proximal uterus and make the dissection more rapid and targeted [16].

The utilization of ultra-staging techniques in sentinel lymph node biopsy has further optimized nodal assessment by decreasing the complication rate of detecting micrometastases when compared with radical pelvic and/or aortic lymphadenectomy [17, 18]. Studies documenting the anatomy have established that deep injections into the cervix create lymphatic mappings that effectively target the uterine vessels, parametria, lower uterine segment, and cornual areas [18, 19]. Additionally, a meta-analysis demonstrated that the only anatomical site that was significantly associated with an improved ability to identify SLN was the cervical injection [20]. Therefore, a cervical injection is particularly important for successful mapping. The test we conducted is consistent with this practice and supports the usefulness of cervical dye injection for targeting and mapping lymphatic pathways from the uterus. The anatomical locations where we identified SLNs, primarily in the obturator and external iliac regions, were consistent with the known lymphatic drainage pathways of the uterus, as documented in large multicenter studies [21].

Our findings on the feasibility of SLN mapping via V-NOTES align with the growing body of literature that supports this approach. Seminal studies by Lee et al. (2022) have established V-NOTES as a viable option for endometrial cancer staging. Our study contributes to this field by specifically comparing two different tracer agents within the V-NOTES framework, a question not extensively addressed in initial feasibility studies. The successful SLN detection rates we achieved were comparable to those reported in these foundational V-NOTES papers, further strengthening the argument for its adoption in gynecologic oncology [12].

This study compared two staining methods, ICG and MB, in women undergoing V-NOTES staging surgery for endometrial cancer. The groups were homogeneous in terms of baseline demographic and clinical characteristics, including BMI. Previous evidence suggests that BMI may be inversely related to SLN mapping success and may influence the choice of the dyeing agent. More specifically, ICG has been reported to perform more successfully in patients with higher BMI and lower body BMI owing to better tissue penetration and fluorescence, which may enhance the identification of lymphatics in visceral and retroperitoneal fat environments. However, the current study was not designed to address the direct effects of BMI; rather, the patient groups were purposefully matched to account for this potential confounder in the analysis.

Our results showed that ICG had better SLN detection rates and greater average SLNs identified per patient than MB, which is consistent with previous investigations that demonstrated the benefits of using real-time fluorescence imaging for lymphatic mapping. This likely aided in the easier and accurate identification of SLNs by allowing for clear visualization of lymphatic flow. Although the rate of bilateral identification was greater in the ICG group (80.0% vs. 65.0%), it did not reach statistical significance. Clinically, this is meaningful because the accurate identification of bilateral mapping is important for comprehensive nodal staging, particularly given the complex and often asymmetric nature of pelvic lymphatic drainage. Larger multicenter studies are required to confirm this possible advantage. While the identification rates of SLN varied, there were no differences in surgical times, fluid losses, or rates of complications for adverse events between groups, suggesting that ICG does not negatively impact the safety of V-NOTES. Moreover, it is worth noting that the time to first identify the SLN was not significantly different between the groups, indicating that the addition of real-time fluorescent imaging to each of the case workflows did not add significant time to the cases.

The histopathologic results showed that the rates of SLN with metastasis were similar in both groups (21.1% for ICG vs. 21.2% for MB), which demonstrates a similar sensitivity for detecting metastatic disease [14]. Furthermore, the overall rate of SLN identification as greater for ICG, in totality, this likely indicates a lower false-negative rate, which provides at least some assurance that nodal status is ascertainable.

In response to the reviewer’s insightful suggestion, we performed a subgroup analysis to identify factors associated with the need for complete lymphadenectomy. Our findings indicate that the primary drivers for performing full lymphadenectomy were adverse pathological features, such as high tumor grade, rather than technical failures of the mapping technique itself. This is a clinically reassuring finding, suggesting that surgical decisions are appropriately guided by established oncological principles, where higher-risk tumors warrant a more extensive nodal evaluation. This analysis further strengthens the reliability of the SLN mapping procedure in the low-risk population, as these patients safely spared the morbidity of full lymphadenectomy.

Although numerous studies have compared SLN biopsy using ICG and blue dye in laparoscopic or robotic surgery, a growing body of evidence points to the superiority of fluorescence-guided mapping. The superior detection rate of ICG in our V-NOTES series aligns with findings from other minimally invasive platforms, where ICG has consistently outperformed blue dye in both laparoscopic and robot-assisted surgeries [22]. Our study contributes to the ongoing discussion by demonstrating these advantages specifically within the unique V-NOTES approach, further solidifying the role of ICG as the preferred tracer for minimally invasive endometrial cancer staging.

### Limitations and strengths

This study had several limitations. First, it was a single-center study, and external validity may have limited generalizability based on surgeons’ expertise, demographics of the patients, etc. Second, due to the small sample size, we acknowledge that the statistical power to compare differences in secondary outcomes, including complication and bilateral SLN identification rates, was limited. Furthermore, we report only short-term follow-up since we were unable to evaluate the oncologic outcomes (recurrence rates and overall survival) with an adequate follow-up duration. The study was also limited because we did not account for potential differences in mapping rates based on BMI when using the two different agents. Finally, there was no cost-effectiveness analysis in this study, which may be relevant in light of ICG’s role as a new standalone dyeing agent with potential for use during V-NOTES procedures.

A primary limitation of our study was that the true false-negative rate for SLN mapping could not be calculated, as completion lymphadenectomy was not performed on all node-negative patients. This approach is consistent with the current clinical guidelines aimed at reducing surgical morbidity. Another significant limitation is the short median follow-up duration of only 8 months, which is insufficient to make definitive conclusions regarding long-term oncologic outcomes. Therefore, the absence of nodal recurrence in our cohort should be cautiously interpreted. However, it is important to note that PET scans performed at the 6-month postoperative mark also showed no evidence of local or distant recurrent metastasis or lymph node metastasis.

A limitation of our study is that the analysis was confined to successfully completing the V-NOTES procedures. Patients requiring conversion to standard laparoscopy were not included, indicating that our findings on surgical outcomes may not be generalizable to more complex cases. The conversion rate and reasons, but not the focus of this comparative dye study, are important considerations for the overall feasibility of this advanced surgical technique.

Another limitation is the unavailability of intraoperative frozen section (FS) analysis of SLNs. However, while FS was not available at our institution owing to logistical constraints, our decision-making process was not based solely on the surgeon’s subjective assessment. Instead, we employed a two-stage evaluation process to enhance predictive accuracy. The first stage consisted of comprehensive preoperative staging with PET/CT for all patients, providing detailed information on lymph node status and potential metastases. In the second stage, this high-accuracy preoperative data robustly informed the surgeon’s intraoperative assessment, thereby guiding the decision to complete lymphadenectomy. This compensated approach, which is utilized in many centers facing similar constraints, allows for reliable oncological staging and a safe surgical procedure. The results of this study should be interpreted within the limitations described, but there are also some notable strengths. This may be the first trial to investigate ICG versus methylene blue in SLN mapping of V-NOTES procedures and to provide important initial data for this new surgical technique. The study included a standardized surgical protocol, real-time fluorescence imaging for intraoperative SLN assessment with ICG, which improved the accuracy and consistency of SLN mapping. Furthermore, a relatively homogenous patient cohort may have limited confounders and may optimize the internal validity of the results. Lastly, the study highlights ICG’s positive effect of ICG on achieving increased overall SLN mapping rates, which may reduce false negative results and improve inconclusive accuracy to achieve better surgical staging [23].

## Data Availability

The data that support the findings of this study are available from the corresponding author, upon reasonable request.
